# The Preterm Heart-Brain Axis in Young Adulthood: The Impact of Birth History and Modifiable Risk Factors

**DOI:** 10.3390/jcm10061285

**Published:** 2021-03-19

**Authors:** Winok Lapidaire, Chris Clark, Mary S. Fewtrell, Alan Lucas, Paul Leeson, Adam J. Lewandowski

**Affiliations:** 1 Oxford Cardiovascular Clinical Research Facility, Division of Cardiovascular Medicine, Radcliffe Department of Medicine, University of Oxford, Oxford OX3 9DU, UK; paul.leeson@cardiov.ox.ac.uk; 2 UCL GOS Institute of Child Health, University College London, London WC1N 1EH, UK; christopher.clark@ucl.ac.uk (C.C.); m.fewtrell@ucl.ac.uk (M.S.F.); a.lucas@ucl.ac.uk (A.L.)

**Keywords:** preterm, cardiac, brain, young adult, MRI, heart-brain axis

## Abstract

People born preterm are at risk of developing both cardiac and brain abnormalities. We aimed to investigate whether cardiovascular physiology may directly affect brain structure in young adulthood and whether cardiac changes are associated with modifiable biomarkers. Forty-eight people born preterm, followed since birth, underwent cardiac MRI at age 25.1 ± 1.4 years and brain MRI at age 33.4 ± 1.0 years. Term born controls were recruited at both time points for comparison. Cardiac left and right ventricular stroke volume, left and right ventricular end diastolic volume and right ventricular ejection fraction were significantly different between preterm and term born controls and associated with subcortical brain volumes and fractional anisotropy in the corpus callosum in the preterm group. This suggests that cardiovascular abnormalities in young adults born preterm are associated with potentially adverse future brain health. Associations between left ventricular stroke volume indexed to body surface area and right putamen volumes, as well as left ventricular end diastolic length and left thalamus volumes, remained significant when adjusting for early life factors related to prematurity. Although no significant associations were found between modifiable biomarkers and cardiac physiology, this highlights that cardiovascular health interventions may also be important for brain health in preterm born adults.

## 1. Introduction

Each year approximately 15 million infants are born prematurely [1]. The disruption of developmental processes caused by preterm birth can have long-term consequences. Abnormalities in the heart and the brain have been independently reported in people born preterm from infancy through to young adulthood [2,3]. People born preterm have altered cardiac structure and function [4,5] as well as higher peripheral arterial stiffness [6]. In the brain, consequences of preterm birth are primarily characterised as lower integrity of the white matter [7,8,9], which also contributes to secondary neuronal degeneration [10], causing a reduction in subcortical volumes [11,12,13,14]. Importantly, the pathways most vulnerable to dysmaturation will also predispose to further injury and prevent repair [15].

Until now, the effects of preterm birth on both cardiac and brain outcomes have been investigated separately. The underlying assumption is that early life factors associated with preterm birth, for example, the effect of early exposure to the extrauterine environment or injury during infancy [16], affect both organs independently. However, it is also possible that cardiovascular disease across the life span leads to abnormalities in the developing and adult brain [17]. 

The link between cardiovascular and brain health has been observed in the general population in mid- to later life [18,19,20,21]. Left ventricular (LV) dysfunction may result in lower perfusion in the brain, hindering development and potentially accelerating neurodegeneration, resulting in lower brain volumes and lower white matter integrity [18,19,20,21,22], while right ventricular (RV) dysfunction results in suboptimal perfusion into the lungs and thus lower oxygenation and inability to maintain cardiac output during exercise [23]. Reduced oxygen availability in the brain can result in white matter damage [24]. In addition, vascular stiffness and high blood pressure have been associated with white matter damage [18,25]. The corpus callosum, the internal capsule and the corona radiata may be the most vulnerable regions [25]. In people born preterm, brain structure is particularly vulnerable and cardiac alterations have developed since the start of life. Preterm infants and infants with congenital heart disease show signs of a white matter dysmaturation disorder, which could explain how even those who survive without major sequelae can have neurodevelopmental deficits [26]. The window of opportunity for repeated injury thus covers their whole lifespan and can likely be detected much earlier than in the general population. The corpus callosum, the internal capsule and the corona radiata already show vulnerability in preterm infants around term-equivalent age [27]. Similarly, lower subcortical volumes (thalamus, hippocampus and putamen) are already observed in preterm infants around term-equivalent age [28,29], but this suboptimal development could be exacerbated by cardiovascular dysfunction [30].

Although there are reports of both cardiac and brain abnormalities by adulthood in people born preterm, these are often studied in different cohorts and can therefore not be directly linked. Furthermore, there is a paucity of research on the long-term impact of premature birth due to limited survival rates of preterm infants prior to the 1980s and the dearth of long-term follow-up of preterm cohorts. Improvements in preterm infant care have boosted survival rates of preterm infants, but cardiovascular abnormalities remain common in people born preterm [3]. As the first large cohorts of surviving preterm infants have now reached young adulthood, it is important to investigate and potentially mitigate the effect cardiovascular abnormalities may have on impaired neurodevelopment and potential later neurocognitive decline [17].

This study aims to investigate if cardiac alterations in young adults born preterm can have consequences for the brain. This will be done in the following steps: (1) identifying cardiac alterations in young adults born preterm as compared to term born controls, (2) investigating in the preterm group whether these cardiac alterations are associated with brain structures five to 10 years later and (3) investigate whether brain structure associated with cardiac alterations in step 2 are “abnormal”, i.e., different between preterm and term born young adults. In addition, potential targets for intervention to protect long-term health will be identified by examining whether the cardiac alterations are associated with modifiable biomarkers in young adulthood.

## 2. Materials and Methods

### 2.1. Demographics

Between 1982 and 1985, 926 infants with birth weights below 1850g without major congenital abnormalities were recruited for a nutritional intervention trial from five paediatric neonatology units in England (Cambridge, Ipswich, King’s Lynn, Norwich and Sheffield) [31]. Information was recorded prospectively by research staff during the hospital stay on the infant (gestational age, birthweight, sex, the Apgar score at 5 min, administration of steroids (yes/no), number of days on ventilation, recorded infection confirmed by a positive blood culture and the percentage of mother’s milk in their diet) as well as on the mother (hypertension during pregnancy (yes/no), current smoking (yes/no) and education level (no qualifications/O-levels or GCSEs/A-levels and/or university or post-school training). 

Of those subjects, 102 participated in a follow-up study for detailed cardiovascular phenotyping, including cardiac MRI, between 22 and 28 years of age. In addition, 102 age and sex frequency matched young adults (aged 20–30 years) born at term to uncomplicated pregnancies were recruited from the general population as a comparison group for this population [5]. Both preterm and control participants were screened to exclude those with congenital abnormalities.

At age 31–35 years, 71 participants from the original infant cohort were followed up for detailed brain MRI. In addition, a new sample of 72 term born control subjects aged 25–40 years with no history of neurological disease was recruited from the general population as a comparison group for this study.

**Figure 1:**
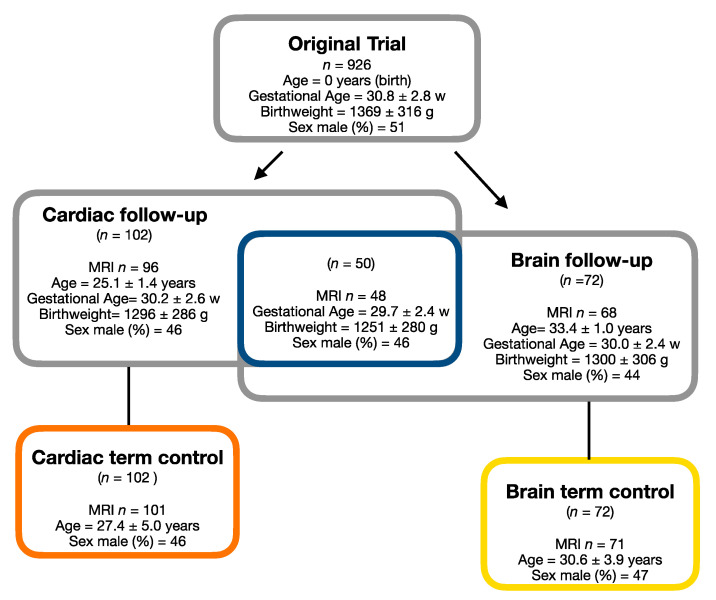
Participant flow chart.

Fifty of the preterm subjects were included in both the cardiac and the brain MRI follow-up studies (Figure 1). The data from this preterm group is used in all analyses in this study.

### 2.2. Cardiac Follow-Up

At the cardiovascular follow-up, waist and hip circumference as well as blood pressure were recorded. The last two out of three brachial blood pressure measurements recorded on the left arm (HEM-705CP, OMRON, Tokyo, Japan) were averaged for analysis. Aortic blood pressure was assessed by left radial artery applanation tonometry to derive ascending aortic pressure waveforms (SphygmoCor Analysis System, Sydney, Australia). In addition, a blood sample was taken from which cholesterol (total, HDL and LDL), insulin, glucose and triglyceride levels were measured. Self-reported current smoking behaviour was collected via a questionnaire. Cardiac MRI was performed on a 1.5-T Siemens Sonata scanner (Siemens Medical Solutions, Erlangen, Germany). Following acquisition of pilot images of the cardiac anatomy, long-axis steady-state free precession cine images (echo time: 1.5 ms; repetition time: 3.0 ms; flip angle: 60°) were acquired. These were then used to plan and acquire a steady-state free precession short-axis cine stack aligned to the LV to obtain coverage of the entire LV and RV for each participant (slice thickness of 7 mm and an interslice gap of 3 mm). All cardiac MRI was prospectively ECG-gated with a pre-cordial 3-lead ECG and acquired during end-expiration breath holding. Parameters reflecting LV and RV volumes and function were extracted from the images (Argus, Siemens Medical Solutions, Erlangen, Germany). LV and RV volumes were indexed to body surface area. LV and RV stroke volumes were calculated by subtracting end systolic volumes from end diastolic volumes, and ejection fractions were calculated by dividing the stroke volume by the end diastolic volume ×100%. LV global longitudinal systolic strain was quantified with TomTec 2D Cardiac Performance Analysis MR (TomTec Diogenes, Munich, Germany). The software tracked the motion of related features adjacent to the endocardial border over the cardiac cycle to produce endocardial strain parameters. 

### 2.3. Brain Follow-Up 

Brain MRI was performed on a 3T Siemens Prisma scanner (Siemens Medical Solutions, Erlangen, Germany) using a self-shielding gradient set with maximum gradient strength of 80 mT m^−1^ and a 64 channel quadrature head coil. T1-weighted volumes were acquired with 1mm isotropic voxels (echo time: 2.74 ms, repetition time: 2300 ms). The T1-images were also processed using the FMRIB Software Library (FSL) [32,33]. The FSL Anat pipeline was used for brain extraction and FSL’s FIRST tool for parcellation (ventricle, thalamus, hippocampus and putamen on both sides of the brain) [33]. Multi-shell diffusion MRI was acquired with 2mm isotropic voxels using multi-band echo-planar diffusion-weighted images with an optimized two-shell protocol: two 60-direction shells of b = 1000 s mm^−2^ and b = 2200 s mm^−2^, interleaved with 14 T2-weighted (b = 0) volumes (echo time: 60 ms, repetition time: 3050 ms). FSL was also used to fit a diffusion tensor and create tensor maps [34]. Subsequently, probabilistic neighbourhood tractography, an automated method for extraction of major white matter tracts using reference tracts as a topological guide [35], was used to delineate white matter tracts. As the study sample only included young adults without major morphological brain abnormalities in the white matter (such as large lesions, which were checked for visually), a pretrained model was used for the reference tracts. Fractional anisotropy (FA) values were averaged across the voxels of tracts in each participant (corpus callosum, left and right internal capsule, and left and right corona radiata).

### 2.4. Statistical Analysis 

**Figure 2:**
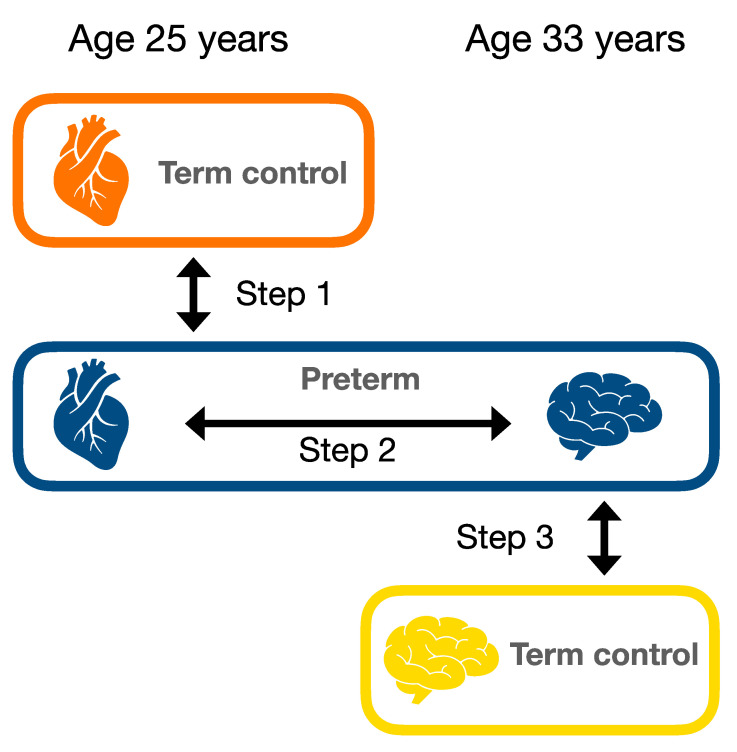
Analysis steps. Steps are presented in the order of the study objectives: (**1**) identifying cardiac alterations in young adults born preterm as compared to term born controls, (**2**) investigating in the preterm group whether these cardiac alterations are associated with brain structures five to 10 years later and (**3**) investigate whether the brain structures associated with cardiac alterations in step 2 are different between preterm and term born young adults.

The mean and standard deviation (SD) are presented for the parameters within each group. Group differences between preterm and term born participants (step 1 and 3 in Figure 2) were examined with an analysis of covariance (ANCOVA). The relationships between cardiac and brain outcomes in the preterm group (step 2 in Figure 2) as well as the analyses between modifiable late life factors and cardiac outcomes in the preterm group were examined with general linear regression analysis and presented as the unstandardised β coefficient and corresponding 95% confidence interval (CI). *p*-values < 0.01 were considered to be statistically significant.

Sex was included as a covariate in all analyses. Age at the time of the brain scan was added as a covariate in analyses with a brain parameter as the dependent variable, while age at the time of the cardiac scan was included as a covariate in analyses with a cardiac variable as the dependent variable. For analyses adjusting for early life factors (gestational age, birth weight, days of ventilation, patent ductus arteriosus, Apgar score at 5 min, steroids, infection, percentage of mother’s milk in diet, hypertensive pregnancy, maternal smoking and maternal education), the parameters that were significantly associated with either the dependent or the independent variable, when adjusted for age and sex, were included as covariates. 

## 3. Results

### 3.1. Demographics 

Ninety-six preterm born subjects and 101 term born subjects had cardiac MRI scans of sufficient quality for analysis. Sixty-eight preterm subjects and 71 term born subjects had brain MRI scans of sufficient quality for analysis. Forty-eight subjects had both cardiac MRI and brain MRI scans of sufficient quality for analysis. One out of the 48 preterm subjects did not have central pulse pressure or blood pressure measures. Participant characteristics for those participants with MRI scans of both their heart and brain are summarized in Table 1. The mean age was 25.1 (±1.4) years at the cardiac follow-up and 33.4 (±1.0) years at the brain follow-up. The preterm and term groups at the cardiac follow-up did not differ significantly in age (t = −0.32, *p* = 0.75) or sex (χ^2^ = 0, *p* > 0.99). At the brain follow-up, there was a significant difference in age (t = −5.25, *p* < 0.001), but not in sex (χ^2^ = 0.01, *p* = 0.91) between the preterm and the term group.

### 3.2. Step 1: Cardiac—Preterm/Control

Table 2 shows differences in cardiac features derived from cardiac MRI scans between young adults born preterm compared to young adults born at term. Young adults born preterm have lower LV stroke volume and end diastolic volume, lower LV global longitudinal peak systolic strain, lower LV length, lower RV end diastolic volume and stroke volume, higher aortic stiffness, higher central systolic blood pressure and higher central pulse pressure. There were no differences in LV ejection fraction between groups.

### 3.3. Step 2: Cardiac/Brain—Preterm

Of the parameters that were significantly different between preterm and term born groups, an increase of 1 mL/m^2^ indexed LV stroke volume was associated with a 31.8 (95% CI: 8.0, 55.5) mm^3^ increase in right putamen volumes (*p* = 0.010). An increase of 1 mm in LV end diastolic length was associated with an increase of 707.1 (95% CI: (248.5, 1165.8) mm^3^ left thalamus volume (*p* = 0.003). An increase of 1% RV ejection fraction was associated with a decrease of 278.1 (95% CI: −484.9, −71.3) mm^3^ in left brain ventricle volume (*p* = 0.01) as well as with an increase of 0.003 (95% CI: 0.001, 0.004) in FA in the corpus callosum (*p* = 0.002). A 1 mL/m^2^ indexed RV stroke volume was also associated with an increase of 0.002 (95% CI: 0.0007, 0.004) in FA in the corpus callosum (*p* = 0.004), and a 1 mL/m^2^ indexed RV end diastolic volume was associated with a 19.6 (95% CI: 5.3, 33.8) mm^3^ right putamen volume (*p* = 0.008).

**Figure 3:**
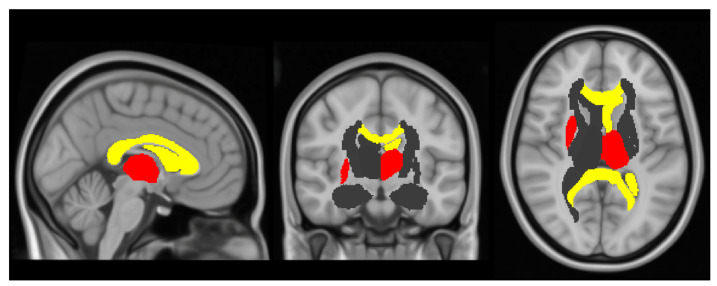
Relationship between cardiac and brain MRI parameters. Brain regions that showed significant relationships with cardiac structure or function adjusted for age and sex (yellow), and additionally for early life factors (red). Areas with no significant relationships with cardiac variables are shown in grey.

The associations between indexed LV stroke volume and the right putamen (β = 31.8, 95% CI: 8,55.5, *p* = 0.01) and LV end diastolic length and left thalamus volumes (β = 664.6, 95% CI: 191.8,1137.4, *p* = 0.007) remained significant at *p* < 0.01 when adjusting for the early life factors that were significantly associated with either the cardiac predictor or the brain outcome variable (Figure 3).

There were no significant associations between modifiable late life factors (current smoking, waist to hip ratio, cholesterol level, HDL, LDL, insulin, glucose and triglycerides) and indexed LV stroke volume and LV end diastolic length when adjusted for age at cardiac scan, sex and early life factors that were significantly associated with the cardiac predictor.

### 3.4. Step 3: Brain—Preterm/Control

Left brain ventricle volume, left thalamus, right putamen and FA in the corpus callosum were significantly associated with cardiac parameters. Compared to term born controls, the left ventricle is significantly larger (*p* = 0.005) and the left thalamus significantly smaller (*p* < 0.001) in the preterm born participants, as shown in Table 3.

## 4. Discussion

This is the first study directly linking cardiac structure and function with future brain structure in preterm born young adults. Associations between LV stroke volume indexed to body surface area and right putamen volumes as well as LV end diastolic length and left thalamus volumes remained significant when adjusting for early life factors related to prematurity.

In concurrence with the literature and previous reports of this cohort, this study indicates that people born preterm have alterations in cardiac function as compared to people born at term. The combination of lower end diastolic volume and stroke volume with preserved ventricular ejection fraction on the left side of the heart [5] and lower end diastolic volume, stroke volume and ventricular ejection fraction on the right side of the heart [4] has previously been reported for the full cohort of 102 preterm-born young adults. This, in combination with impaired LV systolic and impaired RV response to moderate and high intensity physiological stress seen in other preterm populations, suggests a reduction in myocardial functional reserve [36,37,38]. As previously described, these preterm participants have been found to have higher central pulse pressure and systolic blood pressure [4,5]. In addition, this study reports lower RV and LV end diastolic length and higher arterial stiffness (pulse wave velocity) in the preterm group as compared to the term group.

In the current study, we found significant associations between altered cardiovascular parameters and brain parameters measured five to 10 years later. Although causality cannot be established, these findings are in line with patterns observed in older adults, where a wealth of research has reported relationships between cardiovascular and brain health. For instance, cardiac changes have been associated with current and future brain changes [39], vascular risk factors have been associated with reduction in current and future brain volume [40], increased blood pressure has been linked to reduced white matter integrity [18], increased aortic stiffness to altered brain structure and function [19], left atrial hypertrophy to reduced white matter integrity in midlife [20] and reduced cardiac index to larger ventricles. 

The mechanisms underlying the relationships between cardiac and brain outcomes in young adults born preterm remain largely elusive. It is possible that both cardiac and brain alterations have developed since birth. Preterm birth occurs at a time of rapid development of both the heart and brain, and any disturbance during this critical phase can have long-term consequences [41,42,43,44]. In this study, indexed LV stroke volume and end diastolic length and RV ejection fraction and end diastolic volume were associated with higher subcortical and lower brain ventricle volumes. There was also an association between RV ejection fraction as well as indexed RV stroke volume and FA in the corpus callosum. To account for potentially common causes of brain and heart alterations in the early infancy, we adjusted for early life factors. Indexed LV stroke volume and LV end diastolic length remained significant predictors of brain structure, suggesting that not all cardiac–brain alterations in this population group are solely determined by preterm birth itself.

An alternative, yet not mutually exclusive, hypothesis is that the cardiovascular system may play a role in these persisting structural alterations in the brain. A lower ability of the heart to increase its output with increased demand in preterm-born young adults has been shown to lead to reductions in systemic blood flow during exercise [45,46]. This may cause repeated acute hypoperfusion of the brain. Changes in cardiac output, acute or chronic, affect cerebrovascular blood flow (CBF), even when blood pressure remains stable or within the autoregulatory range. Indeed, cardiac output is a key regulator of CBF [47]. CBF reduction precedes cognitive decline and hippocampal atrophy [39], suggesting a negative impact on subcortical brain volumes. Therefore, the association of LV dysfunction with subcortical structures, adjusted for early life factors, in this study supports this hypothesis. Hypoperfusion is associated with white matter damage near the subcortical nuclei in older adults [48], and animal models show particular vulnerability of the hippocampus and corpus callosum [49,50]. Nevertheless, although the effect of the cardiovascular system on the brain is physiologically plausible, this study cannot fully disentangle whether those cardiac–brain relationships that cannot be entirely attributed to early life factors are direct causal relationships or whether these have another common cause.

There was an association between RV ejection fraction as well as indexed RV stroke volume and FA in the corpus callosum. The RV of the heart pumps blood to the lungs and a lower ability of the RV to increase its output with increased demand can result in hypoxic hypoxemia (lower oxygenation of the blood) [51], which could affect the white matter structure [24]. However, we did not observe this relationship when adjusting for early life factors.

If it is the case that not all brain alterations are solely determined by preterm birth and its immediate consequences but could in part be influenced by the cardiovascular system, there may be opportunities to prevent the brain alterations observed in this study. On the other hand, it also bears a warning as this could mean that the brain gets more adversely affected over time in people born preterm due to reduced cardiovascular function. The participants in this study were young, although one in four participants had early stages of hypertension (systolic blood pressure >130 mmHg or diastolic blood pressure >80 mmHg) [52] at the time of the cardiovascular assessment. The proportion of preterm participants classed as hypertensive and the severity of this hypertension are expected to increase with time. In addition, the effects of cardiovascular abnormalities on the brain are likely to become increasingly pronounced with age since the brain is more resilient in adolescence and young adulthood than in midlife and older adulthood. It is therefore critical to understand these early stages and identify potential avenues for early intervention. Although we did not find an association between cardiovascular parameters and a range of modifiable risk factors in young adulthood, future studies should investigate to what extent lifestyle interventions could improve cardiovascular function and whether this also preferentially modifies brain alterations in people born preterm. In addition, it can be explored whether CBF plays a role in the heart–brain links and if these findings have any relevance for cognitive ability.

### Limitations

To disentangle two possible mechanisms for cardiac and brain abnormalities (first, that both cardiac and brain alterations have developed since birth, or second that the cardiovascular system may play a role in these persisting structural alterations in the brain), we statistically adjusted for early life factors in the aim of excluding the majority of their potential impact on these relationships. However, given that the effect on the brain is likely most notable in those subjects with the greatest cardiac dysfunction, which in turn is strongly linked to early life factors, the cardiac–brain relationships may be underestimated when adjusting for early life factors. Note that the heart and brain were not imaged at the same time, and therefore it is not known if the same cardiac features persisted at the time of brain imaging. However, the persistence of these cardiac changes may be inferred based on consistent differences observed across developmental stages [3]. This gap in time is also an advantage of the study, as the effect on the cardiac–brain relationship is likely minimal if the causes of alterations lie solely around preterm birth, while this time gap is essential to be able to pick up any negative effects that cardiac dysfunction may have on the brain as these effects are often not instantaneous and only become evident with time. In fact, if the aim of the study had been specifically to assess the effect of cardiac abnormalities on brain structure, a longer time gap would have been preferable. In line with this, the cardiac parameters were associated with a statistically significant, yet relatively small decrease in brain white matter structure FA. At this point, such a small decrease is not clinically significant, but could become so over time. Inevitably, the length of this time gap also means that this study is not well suited to discover potential long-term effects of the brain on the cardiovascular system. Future studies should follow-up this cohort to assess the progression of these heart–brain relationships, to determine if other associations emerge and explore whether cardiovascular changes precede brain changes or vice versa. 

## 5. Conclusions

This study reveals that cardiovascular alterations observed in young adults born preterm are associated with brain volume changes approximately five to 10 years later when early life factors associated with preterm birth were accounted for. This highlights the importance of cardiovascular health in relation to future brain health in young adults who are at risk of cardiovascular abnormalities. In addition, it probes further investigation of potential ways to improve cardiovascular health in this population to protect both the heart and the brain throughout adulthood.

## Figures and Tables

**Table 1:** Participant characteristics of the cardiac and brain cohort.

	Cardiac + Brain MRI Cohort (*n* = 48)
Gestational age (weeks), mean (SD)	29.7 (2.4)
Birthweight (grams), mean (SD)	1251 (280)
Days of ventilation, mean (SD)	5.6 (9.7)
Patent ductus arteriosus (%)	27.1
Apgar score at 5 min, mean (SD)	7.9 (1.9)
Steroid use (%)	14.6
Maternal smoking (%)	22.9
Infant infection confirmed by blood culture (%)	6.3
Necrotising enterocolitis confirmed (%)	4.2
Percentage of maternal milk in diet, mean (SD)	48.6 (38.2)
Born to hypertensive pregnancy (%)	27.1
Maternal education level A-levels or higher (%)	22.9

**Table 2:** Cardiac structure and function in preterm born participants who also participated in the brain follow-up study compared to term born controls.

	Preterm Group (*n* = 48)	Term Group (*n* = 101)	Adjusted Mean Difference (95% CI)
LV ejection fraction, %	64.1 (6.6)	64.1 (4.9)	0.0 (−1.9, 1.9)
LV stroke volume, mL/m^2^	44.6 (6.9)	51.3 (8.9)	−6.9 (−9.7, −4.0) **
LV end diastolic volume, mL/m^2^	69.6 (7.8)	80.2 (11.7)	−10.8 (-14.2, −7.4) **
LV end diastolic length, mm	9.1 (0.6)	9.8 (0.7)	−0.7 (−0.9, −0.5) **
LV global longitudinal strain, %	−14.8 (2.9)	−17.9 (4.1)	3 (1.7, 4.4) **
RV ejection fraction, %	56.2 (7.8)	60 (5.3)	−3.7 (−5.8, −1.5) *
RV stroke volume, mL/m^2^	42.9 (9.7)	52.9 (7.2)	−10.1 (−12.8, −7.4) **
RV end diastolic volume, mL/m^2^	75.9 (12.4)	88.5 (11.8)	−12.8 (−16.5, −9.1) **
Pulse wave velocity, m/s	5.9 (0.7)	5.5 (0.6)	0.4 (0.2, 0.6) **
Central systolic blood pressure, mmHg	107.9 (10.9)	97.4 (8.8)	10.6 (7.2, 13.9) **
Central pulse pressure, mmHg	33 (7)	27.8 (5.6)	5.2 (3.1, 7.3) **

Data per group presented as Mean (SD). Analyses are adjusted for sex and age at the time of cardiac MRI. * *p* < 0.01, ** *p* < 0.001.

**Table 3:** Comparison of areas showing significant relationships with cardiac parameters in preterm born participants who also participated in the cardiac follow-up study with to term born controls.

	Preterm Group (*n* = 48)	Term Group (*n* = 71)	Adjusted Mean Difference (95% CI)
Left ventricle, mm^3^	9375.4 (5855.4)	6818.0 (4564.0)	−2949.8 (−923.9, −4975.7) *
Left thalamus, mm^3^	7714.1 (940.8)	8343.1 (922.0)	701.9 (1019.6, 384.3) **
Right putamen, mm^3^	5030.2 (628.2)	5254.4 (560.5)	239.9 (443.6, 36.3)
FA corpus callosum	0.6 (0.1)	0.7 (0.0)	−0.0176 (−0.0349, −0.0002)

Data per group presented as Mean (SD). Analyses are adjusted for sex and age at the time of brain MRI. * *p* < 0.01, ** *p* < 0.001.

## Data Availability

Data available on request due to restrictions on privacy. The data presented in this study are available on request from the corresponding authors.
